# Managing Emotions at Work: A TCCM Systematic Literature Review of Empirical Emotional Labour Research

**DOI:** 10.1002/pchj.70118

**Published:** 2026-08-09

**Authors:** Shuyu Zhang, Michelle Chin Chin Lee, May Young Loh, Stuart C. Carr

**Affiliations:** ^1^ School of Psychology Massey University Auckland New Zealand; ^2^ Psychosocial Safety Climate Global Observatory, School of Psychology Adelaide University Adelaide Australia

**Keywords:** emotional labour, organisational behaviour, systematic literature review, TCCM, workplace wellbeing

## Abstract

Emotional labour (EL) has become a serious concern in the modern workplace due to the rapid development of the service‐oriented economy. Existing literature reviews of emotional labour studies leave many questions unexplored. This study addresses these questions by conducting a holistic review of 143 high‐impact empirical articles published in peer‐reviewed journals from APA PsycInfo, EBSCO Academic Search Premier, Scopus, and Web of Science using the TCCM framework (Theory‐Context‐Characteristics‐Methodology). The document search process is visualised with a PRISMA diagram. We point out several future directions for scholars who are interested in emotional labour. For example, emotional labour research needs to transition from cross‐sectional designs to multi‐wave designs. Most importantly, this study serves as a roadmap for scholars to navigate the complexity of emotional labour research and provides practitioners with evidence‐based strategies to mitigate the negative influence of emotional labour.

## 1 Introduction

In the shift from a manufacturing‐oriented to a service‐oriented global economy, the management of emotions has become as important as physical labour in the current workplace. The centre of this transition lies in what scholars call “emotional labour” (EL), which refers to the behaviour of regulating feelings and expressions to meet interpersonal role expectations at work (Gabriel et al. 2023; Hochschild 1983). While initially conceptualised within the context of traditional service industries (Grandey 2000), it has become a crucial element of any job that requires social interactions (Diefendorff et al. 2006). Empirical evidence suggests that EL is pervasive across diverse professions, from traditional service industries such as education (Ye and Chen 2015), customer service (Johnson and Spector 2007), and healthcare (Jung et al. 2019) to non‐service‐oriented industries such as farming (Aka and Enticott 2025; Qiu et al. 2022) and mining (Guo and Li 2022). Extensive literature identifies EL as a significant factor in well‐being issues, such as burnout (Jeung et al. 2018; Wójcik et al. 2022), strain (van Gelderen et al. 2016), and work stress (Gu et al. 2019). Consequently, EL has emerged as a key subject of inquiry, calling for an extensive understanding of its mechanisms across sectors.

Despite nearly four decades of research and the publication of 16 literature reviews (see Table 1), the current understanding of EL remains segmented rather than holistic. Although existing reviews provide valuable insights, there are a few things they fail to consider:

**TABLE 1:** Summaries of previous literature review of emotional labour in chronological order.

Document	Type of review	Reviewed documents	Keywords
Grandey (2000).	Systematic review	Theories and definitions of emotional labour	Definitions, theories, models, individual differences.
Bono and Vey (2005).	Meta analysis	Empirical emotional labour research	Emotional exhaustion, depersonalization, personal accomplishment, physical complaints, role internalization, self‐esteem, and job satisfaction
Wharton (2009).	Systematic review	Theory and research on emotional labour with a focus on its contributions to sociological understandings of workers and jobs	Emotion management, work, service, interaction, jobs, customers
Hülsheger and Schewe (2011).	Meta analysis	Empirical emotional labour research, with a focus on the relationship of emotional labour and well‐being outcomes	Emotion–rule dissonance, surface acting, and deep acting, well‐being and performance outcomes.
Mesmer‐Magnus et al. (2011).	Meta analysis	Empirical emotional labour research focuses on discordance, harmful consequences (health‐, attitudinal‐, performance‐related)	Emotions and mood, discordance, harmful consequences (health‐, attitudinal‐, performance‐related)
Grandey and Gabriel (2015).	Systematic review	Theories and empirical emotional labour research	Construct development and measurement, chronic and momentary determinants, prediction of employee well‐being, influence on organizational performance, dynamic integration of emotional requirements, emotion regulation and emotion performance.
Humphrey et al. (2015).	Systematic review	Emotional labour research, with a focus on discussing the positive outcomes of emotional labour	Emotional labor; emotional regulation; identification; customer service; leadership
Ye and Chen (2015).	Systematic review	Emotional labour research, with a focus on teacher	Teachers, Emotional Labor, Emotional Display Rule, Emotional Labor Strategy
Edward et al. (2017)	Systematic review and meta‐analysis	Emotional labour research, with a focus on mental health nursing	Burnout; emotional intelligence; emotional labour; mental health nursing; resilience.
Jeung et al. (2018).	Systematic review	Emotional labour research, with a focus on burnout	Emotional labor; burnout; self‐efficacy; type A behavior pattern
Lee and Madera (2019).	Systematic review	Emotional labour research, with a focus on hospitality and tourism	Customer service, Emotional labour, Deep acting, Service performance, Surface acting, Customer satisfaction
Wang et al. (2019).	Systematic review and meta‐analysis	Emotional labour research, with a focus on teacher	Teachers ‘emotional labor, Systematic review, Meta‐analysis, Surface acting, Deep acting, Psychological well‐being
Kariou et al. (2021).	Systematic review	Emotional labour research, with a focus on teacher	Burnout; emotional labor; education; teachers; systematic review
Yang and Chen (2020).	Systematic and meta‐analysis	Theories and research of emotional labour	Emotional labor, service, literature review, meta‐analysis
Gabriel et al. (2023)	Systematic review	Emotional labour research published on *Personnel Psychology*	Emotional labor, emotional performance, emotional regulation, well‐being
Zhao et al. (2025)	Meta‐analysis	Observational studies using integrative approach on emotional labour and mental health	Emotional labor, Deep acting, Surface acting, mental health, meta‐analysis

*Note:* The keywords are directly retrieved from the above publications.

First, the prior literature review suffers from bounded scopes. Specifically, existing reviews tend to be restricted to: (a) Focusing exclusively on single professions (e.g., EL among teachers, e.g., Wang et al. 2019; Ye and Chen 2015, etc.); (b) Narrowing the inquiry to certain relationships (e.g., EL and its association with burnout; Jeung et al. 2018); (c) Examining literature published within certain disciplines (e.g., EL research published in *Personnel Psychology*, Gabriel et al. 2023). While these findings provide a precise understanding of certain topics, they fail to provide a holistic view of EL as a universal workplace phenomenon.

Second, existing reviews that did not suffer from the first limitation (i.e., have a broad scope of literature) were conducted prior to or during the COVID‐19 global lockdown (Bono and Vey 2005; Grandey 2000; Grandey and Gabriel 2015; Yang and Chen 2020). The post‐lockdown workplace has experienced drastic changes such as hybrid work and virtual interactions (Castaneda et al. 2022). Relying on early literature reviews leaves current scholars without an updated understanding of how EL dynamics have adapted to these environmental shifts.

Third, prior literature reviews lack a synthesised framework for future empirical research building. Previous reviews have largely been descriptive summaries rather than structural evaluations. The TCCM framework offers an effective approach as it systematically separates: (a) T—Theory: identifying theoretical stagnation and diversity; (b) C—Context: highlighting industries, cultures, and other aspects of empirical research; (c) C—Characteristics: identifying key variables affecting the studied topic; (d) M—Methodology: exposing the reliance and neglect on certain designs. This framework allows for a systematic identification of theoretical stagnation and methodological gaps that unstructured reviews usually miss.

This review synthesises high‐quality empirical research on EL using the TCCM framework, with the aim of providing a structured and accessible overview of high‐quality EL evidence for both researchers and practitioners. It is designed as an integrative knowledge map of rigorously selected empirical studies, spanning psychology and management research and focusing on full‐time employees engaged in daily interpersonal interactions. By applying clearly defined inclusion criteria centred on journal quality, empirical design, and relevance to EL mechanisms, the review consolidates a curated body of literature into a coherent framework that highlights established findings, cross‐study consistencies, and key conceptual patterns in the field. The synthesis is organised around evidentiary quality, offering a consolidated reference point for understanding the state of EL research and supporting future scholarly and practical developments.

To achieve this objective, we pose the following research questions (RQs) with the assistance of the TCCM framework, and each RQ is subdivided into sub‐questions that have emerged for our analysis:

What are the key concepts and theoretical frameworks adopted by empirical emotional labour research?

What are the contexts for studying emotional labour research?

1. How is emotional labour interpreted across macro, meso and micro perspectives?
2. What role does culture play in emotional labour research?
3. How do family contexts influence the management of emotional labour beyond the workplace?

What are the characteristics of emotional labour research?

3.1 What are the key mediating and moderating mechanisms that dictate the process of emotional labour?

3.2 What are the strategies for addressing the negative consequences of emotional labour?

What are the research designs for conducting emotional labour research?

4.1 How do multi‐wave designs capture the temporal fluctuations of emotional labour?

4.2 How have qualitative investigations contributed to uncovering the lived experiences of emotional labour that quantitative metrics may overlook?

## 2 Methods

### 2.1 Review Method & Framework

The present study conducts a systematic literature review as it addresses issues that a single article may overlook, allowing researchers to have a comprehensive overview of the topic, identify gaps and propose directions for future studies (Bangdiwala 2024). We adopted the TCCM (theory, context, characteristics, methodology) framework to systematically analyse the literature and identify the answers to our research questions. The TCCM framework provides great insights into the theoretical groundwork, contextual applications and methodological guides for future studies (Paul et al. 2023).

### 2.2 Inclusion and Exclusion Criteria

Journal quartile rankings are widely recognised indicators of journal quality within the academic community (Asan and Aslan 2020; Orbay et al. 2020). To ensure the inclusion of high‐quality and high‐impact empirical evidence, one of our inclusion criteria was that only articles published in the first quartile (Q1) journals were considered. The current literature review included articles that met the following inclusion and exclusion criteria: (1) aimed to understand emotional labour and its mechanisms; (2) were published in peer‐reviewed journals; (3) were written in English; (4) included empirical data; (5) included samples of full‐time employees; (6) were published in Q1 journals; (7) presented studies from the areas of psychology (behavioural science) or management. We then manually excluded studies that did not focus on employees with constant exposure to human interactions.

### 2.3 Search Strategy

We conducted the search up to January 2025 in the following databases: APA PsycInfo, EBSCO Academic Search Premier, Scopus, and Web of Science. The following keywords were used during the search: “emotional labour” OR “emotional labor” OR “surface acting” OR “deep acting”. We limited the language to English, and the type of documents was limited to empirical articles published in peer‐reviewed journals. The areas were set to be psychology, behavioural science, management, economics, and business. To ensure the review is comprehensive, there was no time limitation applied, and the search was case‐insensitive.

## 3 Results

**FIGURE 1:**
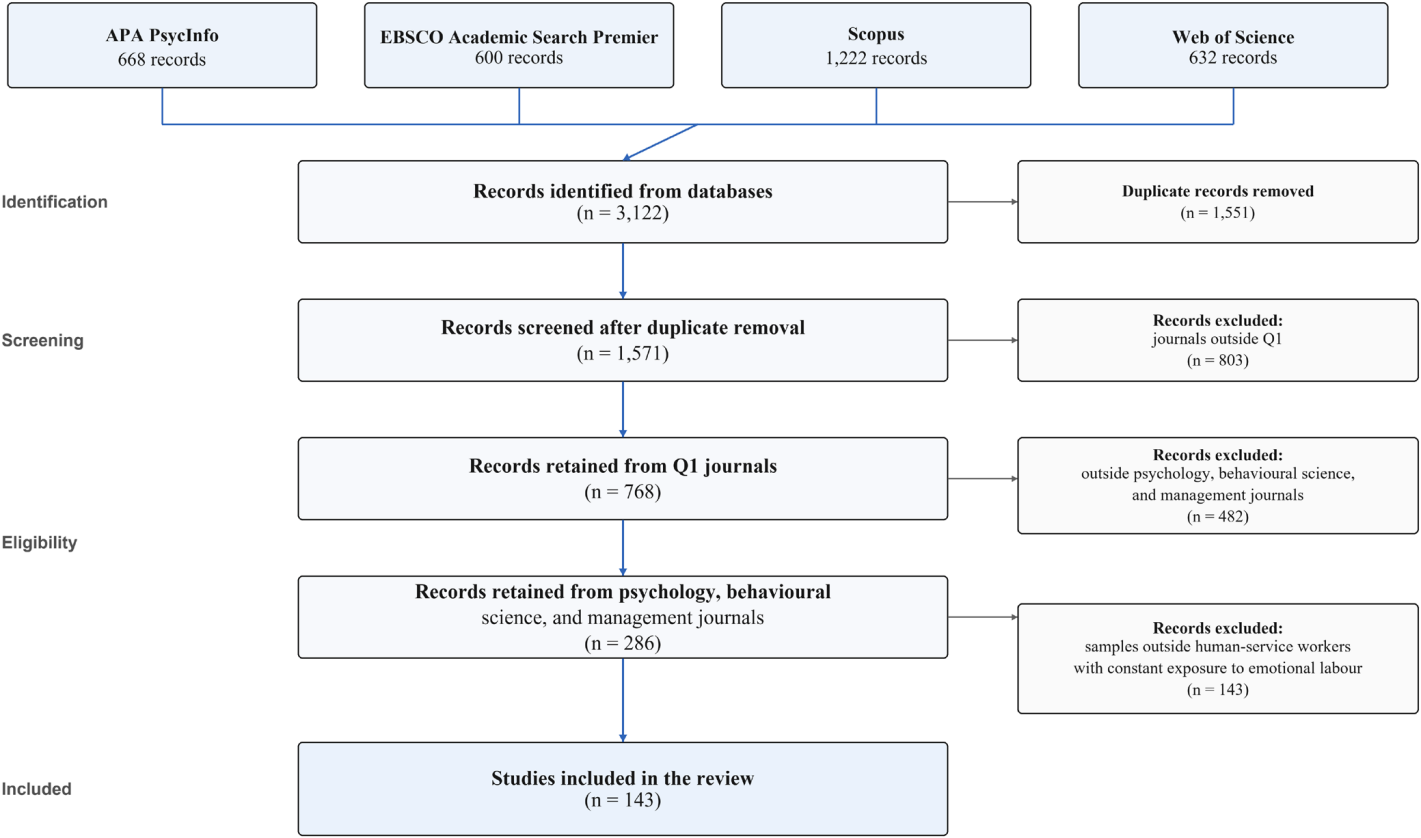
PRISMA diagram of the search process.

A PRISMA diagram was presented to visualise the search process (see Figure 1). We exported the search results from the above four databases and imported them into the literature review software Zotero. Specifically, the initial search included 668 articles from APA PsycInfo, 600 from EBSCO Academic Search Premier, 1222 from Scopus, and 632 from Web of Science. After the duplicate records were removed, we ended up with 1571 articles. Due to the large number of results, we decided to prioritise articles published in Q1 journals, which allows us to concentrate on the rigorously peer‐reviewed studies within the field. Q1 status was determined using the SCImago Journal Rank (SJR) best‐quartile classification for 2025, based on the journal's highest ranked subject category. From the exclusion described above, 768 articles remained. We then further narrowed down the criteria to focus on psychology and management journals only, as these two fields were highly relevant to occupational health. This process left us with 286 articles. In the final round of filtering, we decided to focus on human‐service workers with constant exposure to emotional labour only. By reading through the abstracts of the articles that were left, 143 articles were retained for this systematic literature review.

### 3.1 Publication Trend

**FIGURE 2:**
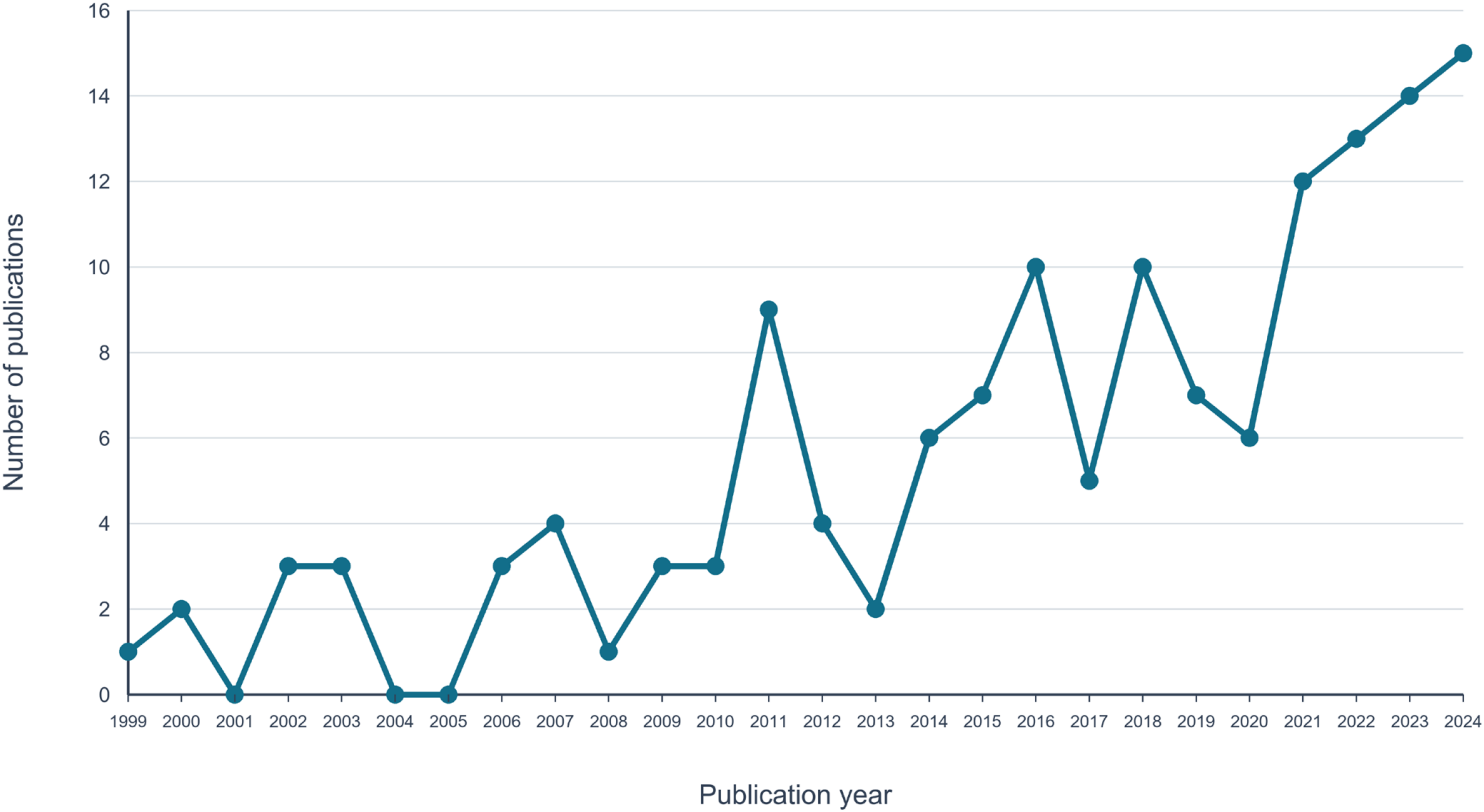
Publication trends.

The publication trend of EL research is displayed in Figure 2. The year of publication ranged from 1999 to 2024. There has been a marked increase in publications of EL after 2020, and the number of publications reached its peak in 2024. This reveals that EL has drawn growing attention from scholars, and we predict that this area will continue to grow according to the upward trend in the figure above.

### 3.2 Journal Contribution

The journal contributions are shown in Table 2. *Journal of Occupational Health Psychology* made the largest contribution to the current database (*n* = 15), followed by *the International Journal of Human Resource Management* (*n* = 11). *Public Personnel Management, Review of Public Personnel Administration*, and *Journal of Applied Psychology* were jointly ranked third, with six publications each. The publications from the above journals contributed approximately 31% of the articles in the current database.

**TABLE 2:** Journal contributions of the present database.

Journal	Count
Journal of Occupational Health Psychology	15
The International Journal of Human Resource Management	11
Review of Public Personnel Administration	6
Journal of Applied Psychology	6
Public Personnel Management	6
PLoS ONE	5
Journal of Managerial Psychology	5
Personnel Review	4
Work, Employment and Society	4
Journal of Organizational Behavior	4
Educational Psychology	4
The Career Development International	4
Early Education and Development	3
Journal of Service Research	3
European Journal of Work and Organizational Psychology	3
Gender, Work and Organization	3
International Journal of Stress Management	3
Work & Stress	4
Current Psychology	3
Work and Occupations	3
Personnel Psychology	2
International Journal of Mental Health Nursing	2
BMC Psychology	2
Motivation and Emotion	2
Journal of Vocational Behavior	2
British Journal of Criminology	2
Work, Aging and Retirement	2
Stress and Health	2
Social Psychology of Education	2
Sex Roles	2
Australian Journal of Psychology	1
British Journal of Educational Psychology	1
Child & Adolescent Psychiatry & Mental Health	1
Human Resource Management	1
Psychological Inquiry	1
Humanities and Social Sciences Communications	1
Journal of Business and Psychology	1
Death Studies	1
Journal of Health & Social Behavior	1
Frontiers in Psychiatry	1
Psychology of Education Review	1
International Journal of Psychology	1
Comprehensive Psychiatry	1
Learning and Individual Differences	1
Methods in Psychology	1
Mindfulness	1
ELT Journal	1
Computers in Human Behavior	1
Journal of Affective Disorders	1
Journal of Educational Psychology	1
Group and Organization Management	1
Journal of Leadership & Organizational Studies	1
Journal of Positive Psychology	1
Journal of School Psychology	1

*Note:* The present database only included journals that are classified in Q1.

### 3.3 RQ1. What Are the Key Concepts and Theoretical Frameworks Adopted by Emotional Labour Research?

Grandey et al. (2005) suggested that the concept of emotion regulation (Gross 1998b) was the foundation of EL framework. Emotion regulation is the process by which individuals manage the expression, duration or intensity of their emotions, and there are two types of commonly studied emotion regulation strategies: cognitive reappraisal (i.e., antecedent‐focused) or expressive suppression (e.g., response‐focused; Gross 1998a; 2015). There are two types of EL strategies: surface acting (faking the expected emotions) and deep acting (altering emotions to match the expectation; Gabriel et al. 2023). Deep acting was linked to cognitive reappraisal, and surface acting was mapped onto emotional suppression (Grandey 2000). Although these theories are strongly associated (Grandey et al. 2005), there were significant distinctions (Grandey and Gabriel 2015) that deep acting involves cognitive strategies to proactively change one's internal emotional experiences and surface acting is a behavioural modulation that often appears reactively to negative environmental factors. More recently, Grandey and Sayre (2019) advised that EL can be viewed as a real‐world contextualised scenario of emotion regulation strategies.

Another key concept that was utilised repeatedly was display rules (Hochschild 1983), and they referred to the shared beliefs of how emotions should be managed. Empirical evidence advised that display rules are the unit‐level beliefs that guide EL (Diefendorff et al. 2011). Display rules differ by audiences and contexts that for individuals with different levels of social status might be different (Moran et al. 2013). For instance, an employee might be expected to show sympathy when their co‐workers are sick or show happiness when interacting with customers. That is, display rules could be seen as an antecedent of EL, and individuals use emotion regulation strategies to manage their emotions to meet the expectations guided by display rules (Martínez‐Iñigo et al. 2007).

Conservation of Resources (COR; Hobfoll 1989) and the Job Demands‐Resources Model (JD‐R model; Bakker and Demerouti 2007) are the two commonly recruited resources‐based frameworks to explain the negative consequences of EL. COR theory posits that individuals have needs to maintain valuable resources and they would experience negative emotional strains such as burnout when these resources are damaged, lost or threatened (Hobfoll 1989). In this framework, EL could be seen as a workplace stressor that drains resources and negative well‐being outcomes are the consequences of the threat of loss of resources (Park et al. 2014). This theory focuses on analysis at the individual level (Hobfoll 1989). On the other hand, the JD‐R model also emphasises the importance of resources, and it posits that balancing job demands and resources is crucial for employees' outcomes (Bakker and Demerouti 2007). EL was often viewed as a result of high emotional demands from the workplace and low job resources (Gu et al. 2019; Wójcik et al. 2022; Yoo and Arnold 2015), and the exploration could work at both individual and organisational levels (Demerouti 2025).

Affective Events Theory (AET; Weiss and Cropanzano 1996) advised that individuals' perceptions of, and attitudes toward their working conditions are formed by their emotional responses to specific events occurring at the workplace rather than general judgements about job characteristics. AET provides a framework for understanding EL and EL could be seen as a specific type of affective event, where the demand to manage emotions could trigger negative emotional reactions if it conflicts with one's genuine emotions (Rupp and Spencer 2006; Kinman 2009). This theory was often adapted to understand the precursors to EL (Grandey 2000). However, recent research used this theory to explore the indirect effects of EL. For example, the effect of within‐person variation in affective arousal could influence the EL process through its impact on empathy (Toomey and Rudolph 2017).

The social exchange theory (SET; Blau 1964) also provides perspectives for understanding EL, which suggests that individuals seek to maximise benefits and minimise costs. Based on SET, EL can be seen as a form of resource investment within employee‐organisation and employee‐customer relationships (Nauman et al. 2022). SET was usually employed as a supporting theory to explain the organisational antecedents of EL, such as perceived organisational support (Mishra 2014; Cho and Song 2017), organisational citizenship behaviour (Nauman et al. 2022), organisational justice (Bechtoldt et al. 2007), etc. When organisations provide adequate support and reward, employees are more likely to engage in deep acting, perceiving the exchange as mutually beneficial. Conversely, when the perceived exchange is not balanced, such as a lack of support, employees are more likely to engage in surface acting (Mishra 2014).

### 3.4 RQ2. What Are the Contexts for Studying Emotional Labour Research?

#### 3.4.1 How Is Emotional Labour Interpreted Across Macro, Meso and Micro Perspectives?

EL is not an isolated individual act but a multi‐level system of antecedents ranging from macro‐cultural norms to meso‐organisational demands/traits to micro‐individual traits.

At the macro level, social norms and cultural frameworks dictate the display rules that govern emotional expression. While display rules generally define the appropriate expression of emotion (Diefendorff et al. 2011; Grandey 2000), their specific content is heavily influenced by cultures. Hofstede's (1980) cultural framework remains the dominant theoretical lens for understanding these variations (Yang and Chen 2020). Although early research was predominantly situated in Western contexts (Nixon et al. 2020; Grandey et al. 2005), it emphasised the need to include cultural antecedents. Subsequent cross‐cultural studies have yielded a consensus: individuals from collectivist cultures (e.g., China, Japan, Korea) are more likely to engage in EL compared to their counterparts in individualistic nations (e.g., the US, UK), largely because suppressing personal emotion to maintain group harmony is a culturally endorsed norm (Allen et al. 2013; Mastracci and Adams 2018; Nixon et al. 2020).

At the meso level, organisations operationalise these macro‐level norms into specific job requirements while simultaneously providing resources to manage them. On one hand, organisations formalise expectations through training programs (Chi and Wang 2018) and emotional climates (Grandey et al. 2012). These mechanisms function as a top‐down process, explicitly teaching newcomers the specific display rules required for customer interactions (Chi and Wang 2018). On the other hand, the organisation provides critical resources that influence how EL is performed. Supervisory support and high‐involvement work practices (HIWPs) have been found to buffer the negative effects of EL, such as burnout and turnover intentions (Chen et al. 2012; Meacham et al. 2022). For instance, organisational support is positively associated with deep acting (a more adaptive strategy) rather than surface acting (Mishra 2014). Furthermore, broader organisational paradigms, such as DEI (Diversity, Equity, and Inclusion) initiatives, can influence the relationship between surface acting and burnout, highlighting the protective role of a supportive organisational environment (Weeks et al. 2023).

At the micro level, individual differences of emotional intelligence and gender determine how employees interpret and enact these emotional requirements. The most extensively researched individual antecedent is emotional intelligence (Austin et al. 2008; Meng et al. 2024). Evidence suggests that individuals with high emotional intelligence possess superior regulation skills, making them less reliant on maladaptive surface acting (Austin et al. 2008) and better equipped to handle emotional demands without sacrificing job satisfaction (Meng et al. 2024). Regarding gender, socialisation patterns frequently impose distinct emotional burdens on women. Female employees are frequently expected to adopt therapeutic roles, whereas male counterparts may adopt more detached educator roles (Polletta and Tufail 2016). Consequently, women often experience more severe negative consequences from surface acting than men (Cottingham et al. 2014; Johnson and Spector 2007). Additionally, personal dispositions play a role; for example, nurses with active characteristics were found to achieve better career success through EL engagement (Han et al. 2022), suggesting that personality traits interact with emotional demands to shape outcomes.

#### 3.4.2 What Role Does Culture Play in Emotional Labour Research?

While globalisation and economic development have reshaped labour markets worldwide, individualism–collectivism is treated as a deeply historically rooted cultural dimension that persists independently of a country's level of economic or institutional development (Akaliyski et al. 2025; Hofstede 1980). This is consistent with a recent study on cultural dimensions (Kashima et al. 2025) that individualism and collectivism are the most extensively researched cultural dimensions, as they were the earliest systematically operationalised and are useful for explaining cognition and emotions. Individualism and collectivism shape the self‐constructs and emotion regulation processes that underlie emotional labour, making it the most theoretically relevant framework in understanding emotional labour (Mastracci and Adams 2018). Accordingly, this section adopts this framework to organise the culture‐related studies in the current pool of literature.

Within this framework, collectivistic contexts (e.g., China, South Korea, Japan) are often associated with a greater emphasis on group harmony and interdependence, where the expression of emotions that may disrupt collective cohesion is discouraged, whereas individualistic contexts (e.g., United States, Canada, Western Europe) tend to emphasise personal autonomy and the legitimacy of open emotional expression (Akaliyski et al. 2025; Moran et al. 2013).

Research comparing the United States and Turkey found that national culture, specifically individualism–collectivism, interacts with organisational display rules to shape EL outcomes (Nixon et al. 2020). Similarly, studies in China, a collectivist society, highlight how social norms encourage positive emotion display and negative emotion suppression (Chen et al. 2012), while research in South Korea examines EL within the context of cultural gender expectations (Yang and Guy 2014).

Research also examines how specific cultural values shape emotional display rules that ultimately influence EL. Some research explored the cross‐cultural comparisons between countries that share similar cultures. For example, Chinese employees were found to engage in deep acting differently than Korean employees, as they showed a stronger mediation effect of self‐regulation between deep acting and job satisfaction (Lu et al. 2020). They directly compared EL strategies between public servants in China and South Korea, explicitly linking their findings to cultural values such as Confucianism and the collectivism–individualism spectrum. This indicates a research focus on moving beyond broad dimensions to understand the role of localised, deep‐seated cultural ethos.

Some research investigated EL within country‐specific contexts and took specific cultural values into consideration, for example, research in India has examined antecedents related to national identity masking and managing customer prejudice (Nath 2011). In Japan, studies have focused on the EL of foreign care workers, shaped by the country's aging population, strict immigration laws, and cultural discrimination (Sever and Tiryaki 2024). Research in Pakistan has explored EL as a job demand within its unique socio‐cultural environment (Nauman et al. 2022).

#### 3.4.3 How Do Family Contexts Influence the Management of Emotional Labour Beyond Workplace?

Research on EL within the familial context consistently demonstrates that emotional regulation within the workplace does not remain isolated to the professional environment but significantly influences employees' home lives, impacting both familial relationships and individual well‐being. Empirical studies indicate that surface acting often results in the depletion of emotional resources. This depletion subsequently leads to increased work–family conflicts and heightened risk of burnout (Montgomery et al. 2006). The emotional demands experienced in the workplace frequently extend into the domestic domain, thereby diminishing individuals' capacity for authentic emotional expression and contributing to emotional exhaustion (Pisaniello et al. 2012). Conversely, when employees engage in deep acting, they tend to experience reduced strain and increased positive emotional spillover. This finding suggests that authentic emotional regulation may serve a restorative function. Collectively, research on EL illustrates that the boundaries between work and family are highly permeable, and that the emotional regulation strategies employed in the workplace can either exacerbate or mitigate strain within the family context (Thomas et al. 2023; Sever and Tiryaki 2024).

### 3.5 RQ3. What Are the Characteristics of Emotional Labour Research?

#### 3.5.1 What Are the Key Mediating and Moderating Mechanisms That Dictate the Process of Emotional Labour?

**FIGURE 3:**
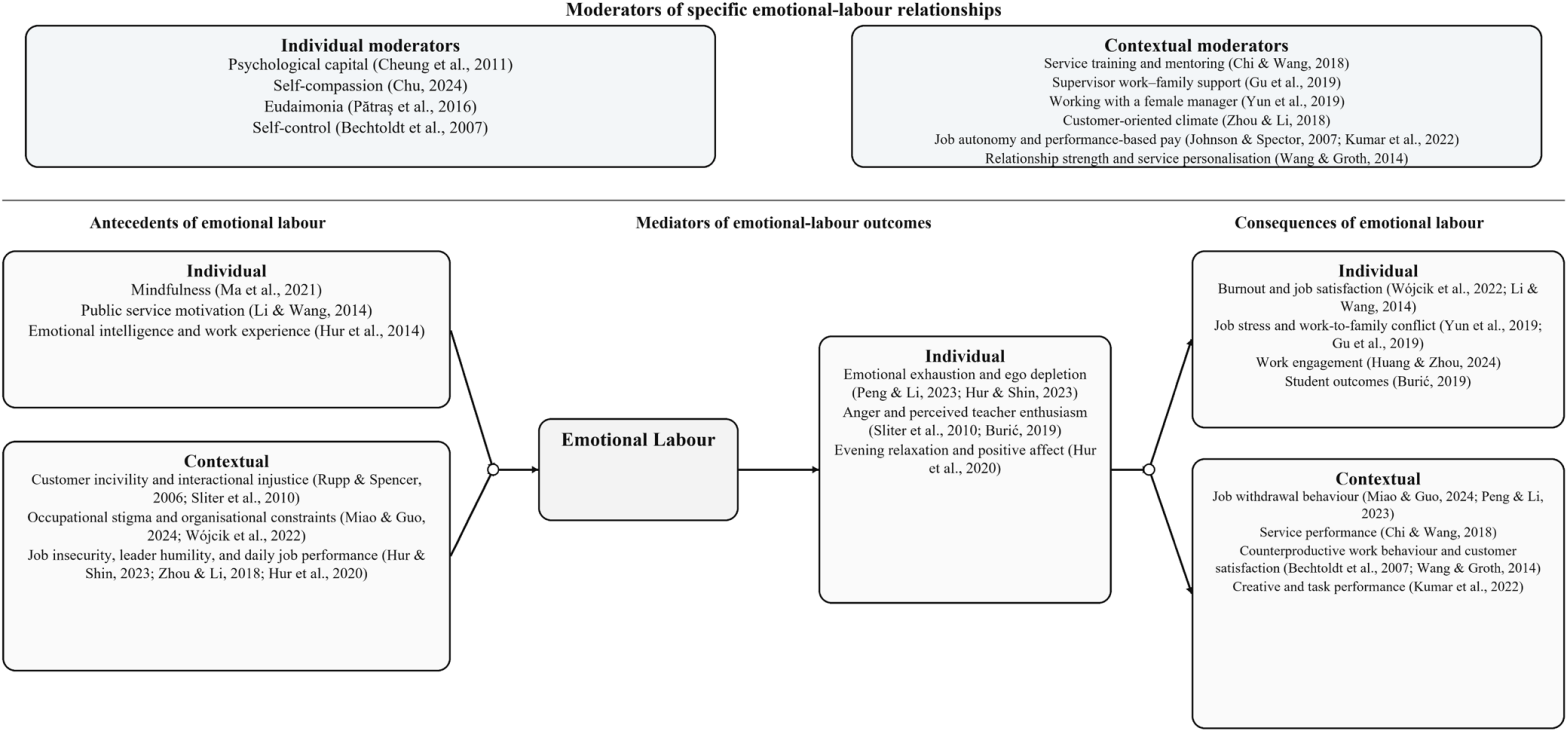
Mediating and moderating mechanisms of emotional labour.

As illustrated in Figure 3, scholars have explored how EL plays different roles in different mechanisms, and the following section maps empirical evidence by grouping the variables into two categories: individual factors and organisational factors. Within each category, we summarise the mediating and moderating mechanisms identified in empirical research.

***Individual‐Driven Mediating Mechanisms.*** Individual characteristics are identified as the primary factor for how EL is performed. Personal resources such as psychological capital and mindfulness have been found to encourage deep acting (Ma et al. 2021). Similarly, EL is a mediator between Public Service Motivation and job satisfaction (Li and Wang 2014). Emotional intelligence serves as a mediator explaining why certain demographics excel at regulation; for example, emotional intelligence mediates the relationship between work experience and the use of deep acting strategies (Hur et al. 2014) and serves as a mechanism that reduces burnout by effectively regulating the labour itself (Guy and Lee 2013). A study conducted among teachers revealed that EL strategies influence work engagement through the mediating role of teacher efficacy; deep acting bolstered efficacy, while surface acting decreased it (Huang and Zhou 2024). Another study reported the negative impact of hiding feelings on student outcomes was fully mediated by a reduction in perceived teacher enthusiasm (Burić 2019). Emotional exhaustion acts as a mediator between EL strategies and withdrawal behaviours (Peng and Li 2023). Daily job insecurity leads to maladaptive EL (higher surface acting and lower deep acting) through the mediating role of ego depletion (Hur and Shin 2023). Daily job performance leads to increased deep acting the following day, a relationship mediated by evening relaxation and next‐morning positive affect (Hur et al. 2020).

***Context‐Driven Mediating Mechanisms.*** Customer incivility and interactional injustice have been shown to increase emotional exhaustion and service difficulty through the mediating paths of EL and anger (Rupp and Spencer 2006; Sliter et al. 2010). Similarly, surface acting mediated the relationship between organisational constraints and nurse burnout (Wójcik et al. 2022). Occupational stigma drives job withdrawal behaviour through a chain mediation involving increased surface acting and decreased deep acting (Miao and Guo 2024). Customer interactional injustice acts as an affective event that triggers anger; anger then partially mediates the relationship between injustice and the increased effort required for EL (Rupp and Spencer 2006).

***Individual‐Driven Moderating Mechanisms.*** High psychological capital weakens the positive association between surface acting and depersonalisation while reinforcing the positive link between deep acting and job satisfaction (Cheung et al. 2011). Similarly, in the nursing profession, self‐compassion moderates the relationship between compassion fatigue and EL, mitigating the detrimental effects of secondary traumatic stress on nurses' ability to regulate emotions (Chu 2024). The relationship between surface acting and exhaustion depends on how employees define well‐being; conversely, for those with strong self‐development beliefs, this relationship is weakened, as they may view emotional regulation as a professional challenge to be managed (Pătraş et al. 2016). Furthermore, self‐regulatory capacities such as self‐control have been shown to moderate the effects of EL on counterproductive work behaviour (Bechtoldt et al. 2007).

***Context‐Driven Moderating Mechanisms.*** Service training and mentoring functions significantly moderate the relationship between EL and service performance (Chi and Wang 2018). To be more specific, service training improves the positive impact of deep acting on performance, while mentoring functions like career development and role modelling can decrease the negative effects of surface acting (Chi and Wang 2018). Leadership style and gender dynamics also play a crucial role. Leader humility facilitates deep acting, though this effect is further moderated by the organisation's customer‐oriented climate (Zhou and Li 2018). Evidence suggested that female managers can buffer the negative effects of EL on job stress, particularly within gendered or hierarchical institutions (Yun et al. 2019). High levels of job autonomy alleviate the negative outcomes associated with EL (Johnson and Spector 2007), while job resources such as supervisor work‐family support moderate the link between surface acting and work‐to‐family conflict (Gu et al. 2019). The effects of surface acting on customer satisfaction are moderated by relationship strength and service personalisation, such that strong employee–customer relationships buffer the negative effects of suppressing negative emotions, whereas highly personalised service contexts intensify them (Wang and Groth 2014). Furthermore, extrinsic motivators such as performance‐based pay moderate the impact of EL on creative and task performance, suggesting financial incentives alter how employees utilise emotional resources (Kumar et al. 2022).

#### 3.5.2 What Are the Strategies for Addressing the Negative Consequences of Emotional Labour?

The solutions to mitigate the negative consequences of EL can be summarised into several categories, and the first is organisational‐level intervention. Organisations have the primary responsibility for creating a work environment that minimises the negative influence of EL. Firstly, organisations are advised to provide training in emotion regulation so that employees engage in more deep acting and use more constructive emotion regulation strategies such as reappraisals and mindfulness rather than expressive suppressions and therefore promote more deep acting behaviours (Grandey and Gabriel 2015; Hülsheger et al. 2010; Pugliesi 1999). Recommended programmes are workshops on cognitive reappraisal techniques, implement training to enhance employees' skills and special training on handling negative customer interactions (Sliter et al. 2010; Williams 2003).

Secondly, organisations should be responsible for enhancing job resources and support systems to increase autonomy, supervisory support, and social support (Chen et al. 2012; Kinman et al. 2011; Lumsden and Black 2018). It is also advised to foster authenticity and genuine emotional expression by creating climates that allow for authentic emotional displays and discourage forced positivity (Hsieh 2012; Mastracci 2021; van Gelderen et al. 2011), improve work design and policies by redesigning jobs to reduce excessive emotional demands, implementing clear, fair emotional display rules and providing breaks and backstage spaces for employees to recover (O'Brien and Linehan 2016; Yue et al. 2015); select and develop emotionally suited employees for the job, using selecting criteria for high emotional intelligence or traits suited for deep acting (Rayner and Espinoza 2015). A practical way to enforce this advice is to recruit psychometrics in the selection process and identify candidates with high emotional intelligence, psychological capital, or traits like agreeableness that facilitate deep acting (Bechtoldt et al. 2007; Cheung et al. 2011).

Individual‐level solutions include adopting healthier emotion regulation strategies by practising deep acting, mindfulness and recovery activities, seeking social and professional support by engaging in social activities, mentoring and debriefing sessions, and developing self‐awareness and reflexivity by reflecting on emotional experiences and aligning emotional expressions with personal and professional values (Burić 2019; Guy and Lee 2013; Song 2021).

Previous research also provided insights into systematic and cultural interventions to these negative outcomes, and they advocated that decision makers should address gendered and structural inequities, challenged gendered EL norms and redistributed work more equally (Franzway 2000; Taylor and Tyler 2000). They also emphasised the importance of promoting ethical and supportive leadership, and leaders who are supportive and foster psychological safety would reduce EL (Chen et al. 2012; Kinman et al. 2011; Lumsden and Black 2018). The use of implementing reflective and compassionate HR practices was another key solution (Brunetto et al. 2022; Grandey et al. 2013).

### 3.6 RQ4. What Are the Research Designs for Conducting Emotional Labour Research?

The current database includes 143 empirical studies: 124 are quantitative, comprising 88 cross‐sectional and 36 multi‐wave studies, while 19 are qualitative. This distribution indicates that empirical EL research is heavily biased towards cross‐sectional, quantitative methods. Although most of the literature reviewed in the preceding section is dominated by these approaches, such designs inherently face two key limitations: challenges with causal inference (Gabriel et al. 2023) and limited provision of rich contextual understanding (Lim 2024). To answer our research question, this section specifically focuses on multi‐wave quantitative studies and qualitative studies. This methodological emphasis is driven not by frequency, but by their unique capacity to address the limitations of cross‐sectional approaches.

#### 3.6.1 How Do Multi‐Wave Designs Capture the Temporal Fluctuations of Emotional Labour?

In our database, 36 studies utilised a multi‐wave quantitative approach. This indicates that EL research was skewed towards cross‐sectional research, and this pattern was in line with the observations of other systematic literature reviews (Yang and Chen 2020; Kariou et al. 2021), and both reviews advised that the field of EL studies would benefit from longitudinal designs. It is necessary to recruit multi‐wave designs to thoroughly examine the causal pathways involved in EL (Geng et al. 2014). Longitudinal data allows us to test whether surface acting and deep acting precede well‐being outcomes (Hülsheger et al. 2010). The time gaps of longitudinal research in our data pool ranged from 1 week (Nauman et al. 2022) to 1 year (Philipp and Schüpbach 2010). EL research with larger time gaps could reveal how EL could impact, or be influenced by different factors in the longer term, and therefore provide better solutions to long‐term well‐being management. For example, Philipp and Schüpbach (2010) conducted a two‐wave study with a time gap of 1 year, and they found that teachers who engage in more deep acting reported significantly less emotional exhaustion after a year. Another study also explored teachers' EL but with a shorter time gap of 5 months, and they found that teachers who reported more emotional exhaustion in the first wave tend to engage in heavier EL in the second wave (Wang et al. 2021). There were also studies that employed shorter time gaps, such as 1 week apart (Andel et al. 2022; Nauman et al. 2022), 2 weeks apart (Becker et al. 2017; Gu et al. 2019), 3 weeks apart (Miao and Guo 2024), etc. These studies are helpful for examining medium‐term processes, such as how EL strategies change unethical behaviours over a few weeks (Nauman et al. 2022). And these studies allow for cross‐lagged panel models testing reciprocal relationships (Gu et al. 2019). Their findings could be beneficial for organisations to improve their well‐being and boost job performance within a shorter period of time (Andel et al. 2022).

Additionally, EL studies that recruited a diary method could capture within‐person fluctuations and dynamic processes (Burić and Wang 2024). Diary studies indicated that EL could change within a day. For example, a diary study conducted among Dutch police officers advised that daily surface acting mediated the association between strain at the beginning of a shift and service performance, as well as the strain at the end of the shift. Their results also indicated that daily surface acting was associated with emotional dissonance if they started a day with high strain (van Gelderen et al. 2016). Another diary study conducted among Canadian teachers investigated EL at both within‐person (i.e., genuine expression of positive emotions was positively associated with student engagement) and between‐person level (i.e., teachers with a stronger trait of enjoyment tended to naturally express positive emotions; Burić and Wang 2024).

To conclude, multi‐wave designs allow researchers to capture temporal dynamics and causal relationships in EL that cannot be explored by cross‐sectional designs.

#### 3.6.2 How Have Qualitative Investigations Contributed to Uncovering the Lived Experiences of Emotional Labour That Quantitative Metrics May Overlook?

In comparison to quantitative studies, qualitative studies have advantages in explaining themes and patterns that are difficult to quantify (Foley and Timonen 2015). However, only 19 out of 143 empirical EL studies in the current database adopted qualitative methods. To answer the research question more clearly, we classified studies into three broad categories: (1) Interview‐based studies that did not articulate a broader methodological framework (e.g., Franzway 2000; Lively 2002; Nath 2011; Polletta and Tufail 2016). (2) Ethnographic studies, which typically rely on fieldwork, and might include participant observations and interviews (Brewer 2023; Lumsden and Black 2018; Taylor and Tyler 2000, etc.). (3) Studies that combined several data collection methods (Sever and Tiryaki 2024; Williams 2003).

Interview‐based studies explored how gender affects EL. A study was conducted among female union officials who continually experience more emotional demands in a male‐dominated movement and adopt shifting strategies to access more political opportunities (Franzway 2000). In another study of the debt‐settlement industry, male agents were able to claim an educational role that eased their emotional dissonance, but female agents were expected to act in a therapeutic role that conflicted with sales expectations and forced surface acting (Polletta and Tufail 2016). Other interviews compared occupational contexts to show how clients and organisations could shape EL. For example, paralegals face intense client‐driven demands, but their substantive involvement may release them from EL (Lively 2002). Human resource staff were exposed to the risk of heavy EL, and organisations should support them in managing demands. Similar findings and corresponding solutions were also proposed for correctional officers who work with incarcerated youth (Perry and Ricciardelli 2021) and hospital staff (Varela Castro et al. 2022).

Ethnographic research allows for in‐depth exploration of EL (Lumsden and Black 2018). However, there were only a few studies that adopted this method. The four ethnographic studies included in our database bring a detailed investigation of EL. An ethnographic study conducted a qualitative comparison of three nursing home facilities, and they argued that EL and organised emotions differ in their presence or absence of organisational display rules and affective requirements (Lopez 2006). Lumsden and Black (2018) ethnographic research of frontline police staff revealed the high EL requirements created by moral expectations, as their job roles included moral duties. More recently, Brewer (2023) conducted a 21‐month hospital ethnography on clinicians, reporting that the desire to avoid engaging in EL can impact patient care and workplace relationships. Research in the airline industry highlighted how managerial pushes for quality service disproportionately increase emotional demands on female staff and the performance of sexual differences while also leaving space for compliance, consent and resistance that are embedded with class relations (Taylor and Tyler 2000).

There were several studies in our database that recruited more than one qualitative method, and they showed the diverse research methods for studying EL. The consensus of the studies of the education industry was that teachers were at high risk of EL, but each study made fresh and unique discoveries. Rayner and Espinoza (2015) conducted a qualitative study using interviews, observations and documentation, and the results showed that performance‐driven reforms have intensified teachers' EL, creating strain when bureaucratic prescriptions conflicted with professional goals. Another case study of teachers' EL analysed the weekly‐reflection statement, teaching documents and interviews of an English teacher, and the results revealed that a teacher's EL might be caused by efforts to manage the negative emotions they felt over how non‐native‐speaker students were treated by other teachers (Song 2021). Another qualitative research of English teachers used Interpretive phenomenological Analysis (IPA; including narratives, focus groups and interviews) reported that teachers managed to gain emotional capital from EL (Ghyasi and Gurbuz 2023). Maynard (2022) conducted a thematic analysis of school staff, using focus group and interviews, and it was reported that managing safeguarding decisions and supporting staff with their emotional experiences at workplace could be beneficial for EL. Additionally, a case study interviewed retail workers indicated that discounters have caused a decline in the complexity and autonomy of sales workers' EL (Ikeler 2016). Foreign female caregivers in Japan experienced heavy EL as they were often perceived as inadequately qualified according to the study of Sever and Tiryaki (2024), and they employed both photovoice and interviews to collect data. Finally, Thomas et al. (2023) conducted co‐constructive patient simulation sessions with child and adolescent psychiatrists and employed thematic analysis to analyse their data. They reported that psychiatrists manage EL by providing safety and containment for both child and parent figures, and their EL also included sustaining difficult family dynamics of their patients.

A study collected data from Australian flight attendants with surveys, and the author advised that they combined both quantitative and qualitative methods as the survey included invitations for open‐ended comments. Their qualitative data indicated that EL could be both positive and difficult at different times for the same person (Williams 2003).

## 4 Discussion

### 4.1 Theoretical Contributions

Based on the TCCM framework, we critically examine empirical EL literature and seek the answers to our research questions. First, this review located the dominant theories and concepts actually recruited in empirical EL research, and they are: emotion regulation (Gross 1998a), display rules (Hochschild 1983), COR (Hobfoll 1989); JD‐R model (Bakker and Demerouti 2007), AET (Weiss and Cropanzano 1996), and SET (Blau 1964). Second, the current review identified the key context for studying EL. There are two broad categories: EL at the workplace, and EL beyond workplace. For EL at the workplace, there are macro, meso and micro perspectives; and EL beyond workplace usually refers to the interaction between work and family, or purely within family context. Third, this review summarised the key relationships that EL was involved in (i.e., mediations and moderations) and recommendations to handle EL. Finally, this review revealed that EL research was predominantly cross‐sectional, and it summarised the highlights and findings of multi‐wave quantitative studies and qualitative studies.

Contributions to a field can be incremental, revelatory, consolidatory, or replicatory (Nicholson et al. 2018). This literature review primarily makes consolidatory contributions by synthesising findings into the cohesive system provided by the TCCM framework. It also makes incremental contributions by systematically aggregating evidence from Q1 journals to offer a comprehensive overview of the field. In addition, by identifying the most applicable theories, we clarify how specific frameworks are operationalised in empirical research. COR and JD‐R have emerged as the dominant theoretical frameworks, but both are most often used to understand the negative consequences of EL. Our findings reveal a persistent bias in EL research towards the dark side rather than its potential positive outcomes, both in the phenomena studied and the theories applied. This concern was raised by Humphrey et al. (2015) a decade ago, and this review supports that this criticism remains relevant. Another incremental contribution is our focused review of multi‐wave and qualitative research, which has received relatively less attention from previous scholars. We highlight the unique insights provided by multi‐wave quantitative and qualitative designs, demonstrating how their findings address critical limitations of the cross‐sectional quantitative research that currently dominates this field.

### 4.2 Practical Implications

The present review provides managerial implications for organisation leaders that are interested in improving employees' mental health by reducing EL. This review highlights the importance of organisational resources such as intervention programmes (Grandey and Gabriel 2015) and workplace supports (Hsieh 2012; van Gelderen et al. 2011). Organisations can design structured training or reflective workshops to help employees manage and regulate EL demands in workplace interactions. These resources are beneficial for employees' adaptive coping strategies, job satisfaction and job performance (Mastracci 2021). Notably, the nature and intensity of EL demands vary considerably across professions (Wang et al. 2019). For example, medical professionals may require targeted support for managing emotional exhaustion in high‐stakes environments, whereas teachers and service workers may benefit from strategies tailored to sustained interpersonal demands and interpersonal interactions. As such, organisations are encouraged to adapt these resources to the specific emotional demands of their occupational context.

This review can also assist employees who are suffering from EL, or counsellors who are keen to help their clients with this issue. Employees can benefit from activities that help with coping and adjusting to the use of EL (Burić 2019; Guy and Lee 2013; Song 2021). Activities such as mindfulness exercises or peer support groups can strengthen their capacity to manage their emotions in adaptive ways and maintain psychological well‐being over time.

### 4.3 Recommendations and Future Directions

***Using a More Positive Theoretical Lens.*** This review reveals that the criticism that EL research is heavily biased towards the dark side (Humphrey et al. 2015) is still relevant after ten years. Future empirical research should consider employing positive theoretical frameworks to explore the positive side of EL. For instance, although JD‐R (Bakker and Demerouti 2007) is one of the dominant theoretical frameworks adopted, few studies have considered its motivational path. Researchers could test models where deep acting behaviours promote work engagement and thrive rather than merely preventing burnout. Similarly, future researchers who employ COR (Hobfoll 1989) as their guiding theory could also consider investigating if EL causes resource gains under certain circumstances. By embracing these positive theoretical lenses, both scholars and practitioners can understand the mechanisms of EL in a more balanced way.

***Investigating bi‐Directional Spillover Effect.*** While existing literature has explored the “work‐to‐family” impact of EL and highlights how emotional experiences at the workplace spill over into their family domains (Montgomery et al. 2006), the reverse path is relatively less investigated. We encourage scholars to explore how one's family dynamics (e.g., family support, parenting stress, etc.) affect their EL usage at the workplace. By shifting from a unidirectional spillover perspective to a bi‐directional relationship, researchers can better understand how resource accumulation or loss in one's personal life dictates the selection and effectiveness of EL strategies in the organisational context. Additionally, more people are working from home or hybrid working (Castaneda et al. 2022) and this new working mode creates new challenges for work‐life balance. Future studies may benefit from examining EL in remote or hybrid settings, where work‐family boundaries may be less clearly defined.

***Using More Qualitative Methods.*** We encourage future scholars to explore EL with more qualitative methods for the following reasons: First, qualitative research is considered as inductive approach and it assumes that reality is a social construct, and all variables are complex and difficult to measure and it has its distinctive power in providing in‐depth insights that enriches the understanding of social phenomena (Lim 2024). When it comes to EL, qualitative approach would be beneficial for understanding the black box of debated issues. For example, in quantitative research, there have been mixed findings about impacts of deep acting (Gabriel et al. 2023). We are optimistic that qualitative approach could bring us fresh and unique explanations to this question. Second, conducting qualitative research does not conflict with quantitative research. There are several studies in our database (e.g., Rayner and Espinoza 2015; Song 2021; Ghyasi and Gurbuz 2023; Maynard 2022, etc.) used combined research methods. These studies not only explore how EL interacts with other variables but also provide deep insights about their participants. Furthermore, qualitative research does not conflict with surveys (i.e., the traditional quantitative data collection format). For example, Williams (2003) used surveys to collect quantitative data and included open‐ended comments. Future studies should consider combining both methods.

***Flexible Time Gaps of Multi‐Wave Research.*** We observe that quantitative research of EL is biased towards cross‐sectional. This is not an uncommon criticism (Jeung et al. 2018; Yang and Chen 2020). However, empirical evidence suggested that EL can be seen as a dynamic process that changes over time (Gabriel and Diefendorff 2015). Most studies adopted time gaps between a week and a year, and we believe EL literature could benefit from more diverse time gaps such as multiple times within a day to capture the fluctuation of its influence within the workday, as the same individual's EL may differ at different time points (Burić and Wang 2024). The findings are helpful for us to understand the nature, changes, and time sensitivity of EL.

## 5 Conclusion

This review maps empirical research on EL published in high‐quality journals. We perform a comprehensive article search and observe the features of the current database. We then address the answers to the research questions we propose at the beginning of this literature review by delving deep into the database. Looking ahead, we identify four priority directions for future research, such as: adopting positive theoretical lenses to examine EL's motivational and resource‐building potential rather than only its depleting effects; investigating the reverse, family‐to‐work spillover of EL alongside the now‐familiar work‐to‐family path, particularly as remote and hybrid working reshape these boundaries; combining qualitative and quantitative methods to illuminate persistently mixed findings, such as those surrounding the effects of deep acting; and diversifying the time gaps used in multi‐wave designs, including within‐day measurement, to better capture how EL fluctuates over the course of a single workday. For practitioners, we also propose suggestions such as targeted resources for managing emotional labour, structured training, and mindfulness exercises that help employees manage EL demands and protect their well‐being. We are optimistic that these findings will help guide scholars working in this field, as well as practitioners seeking to improve workplace conditions and reduce the negative outcomes associated with EL.

## Conflicts of Interest

The authors declare no conflicts of interest.

## Data Availability

Dear editor, The current study is a systematic literature review, and it does not have any empirical data to share. If you need further information, I'm more than happy to assist you.
